# Cloning of transgenic tobacco BY-2 cells; an efficient method to analyse and reduce high natural heterogeneity of transgene expression

**DOI:** 10.1186/1471-2229-9-44

**Published:** 2009-04-22

**Authors:** Eva Nocarova, Lukas Fischer

**Affiliations:** 1 Charles University in Prague, Faculty of Science, Department of Plant Physiology, Vinicna 5, CZ 128 44 Prague 2, Czech Republic

## Abstract

**Background:**

Phenotypic characterization of transgenic cell lines, frequently used in plant biology studies, is complicated because transgene expression in individual cells is often heterogeneous and unstable. To identify the sources and to reduce this heterogeneity, we transformed tobacco (*Nicotiana tabacum *L.) BY-2 cells with a gene encoding green fluorescent protein (GFP) using *Agrobacterium tumefaciens*, and then introduced a simple cloning procedure to generate cell lines derived from the individual transformed cells. Expression of the transgene was monitored by analysing GFP fluorescence in the cloned lines and also in lines obtained directly after transformation.

**Results:**

The majority (~90%) of suspension culture lines derived from *calli *that were obtained directly from transformation consisted of cells with various levels of GFP fluorescence. In contrast, nearly 50% of lines generated by cloning cells from the primary heterogeneous suspensions consisted of cells with homogenous GFP fluorescence. The rest of the lines exhibited "permanent heterogeneity" that could not be resolved by cloning. The extent of fluorescence heterogeneity often varied, even among genetically identical clones derived from the primary transformed lines. In contrast, the offspring of subsequent cloning of the cloned lines was uniform, showing GFP fluorescence intensity and heterogeneity that corresponded to the original clone.

**Conclusion:**

The results demonstrate that, besides genetic heterogeneity detected in some lines, the primary lines often contained a mixture of epigenetically different cells that could be separated by cloning. This indicates that a single integration event frequently results in various heritable expression patterns, which are probably accidental and become stabilized in the offspring of the primary transformed cells early after the integration event. Because heterogeneity in transgene expression has proven to be a serious problem, it is highly advisable to use transgenes tagged with a visual marker for BY-2 transformation. The cloning procedure can be used not only for efficient reduction of expression heterogeneity of such transgenes, but also as a useful tool for studies of transgene expression and other purposes.

## Background

Tobacco BY-2 cell line is the most popular and widely used cell line in plant research. Hundreds of scientific papers have been published using this line as a model to study various aspects of plant cell physiology. BY-2 cells are relatively homogenous, allowing studies of cell phenotypes [1]. Moreover, the cells exhibit high growth rate, enabling synchronization of cell divisions and cell-cycle analyses [2,3]. Being easily transformable either by particle bombardment [4] or by co-cultivation with *Agrobacterium tumefaciens *[5], transgenic derivatives of BY-2 cell line have had high impact in analyses of protein function by ectopic expression, gene knock-outs or translational gene fusions. GFP-tagging of proteins provides viable staining of different cell structures and organelles and analyses of subcellular protein localization [6,7]. The expression of fluorescent protein constructs can be easily monitored by fluorescence microscopy, whereas expression of non-tagged transgenes cannot be readily detected at individual cell level. In both cases, homogenous and stable expression of transgenes is highly desirable for both molecular/biochemical analyses of the total cell culture and for monitoring the effects of transgene expression in individual cells.

Variation in transgene expression in independent transgenic lines has been repeatedly reported to be related to the sequence of the introduced gene construct, involving RNA-sensing mechanism, the locus of insertion, the number of insertion copies, and the initial level of transgene expression [8-13].

The impact of the position of the inserted transgene in the chromosomal environment remains unclear, and the reports are partly controversial. In contrast to classical studies [8], Schubert with colleagues reported that the site of insertion had rather marginal effect; the expression of reporter genes under the control of a strong promoter was comparable among independent transgenic *Arabidopsis *plants harbouring the same transgene copy number [11]. However, silenced transgenes integrated into heterochromatin regions were not included in the study due to selection bias, as revealed by subsequent analyses of transgenic plants or cell lines generated without selection pressure [12,14]. Recently, Fischer with colleagues showed that the integration site significantly influences the sensitivity of the transgene to RNA silencing rather than affecting its initial expression level [15].

In contrast to numerous analyses of independent transgenic lines, much less attention has been paid to analyses of genetically identical clones [16,17], which could bring valuable information about the variability of transgene expression independently of the positional effect.

Analyses of GFP-tagged transgenic BY-2 cell lines in our laboratory repeatedly produced only a low frequency of lines with well-balanced and stable fluorescence in all cells. In order to analyse the nature and sources of this variability, we transformed tobacco BY-2 cell line with a gene encoding free GFP, which allows simple *in situ *evaluation of transgene expression levels *via *assessment of green fluorescence. The homogeneity and stability of GFP fluorescence was monitored in both the primary *calli *obtained after *Agrobacterium*-mediated transformation and in suspension cultures derived from these *calli*. In order to eliminate high natural heterogeneity in *GFP *expression found in the primary lines, we introduced a simple cloning procedure. In addition to reducing the heterogeneity of GFP expression, the method also offered the opportunity to study the variability of transgene expression in genetically homogeneous clones, thus contributing to understanding of the impact of positional effect in transgene expression.

## Results

### GFP fluorescence in primary *calli *and suspensions obtained after transformation

**Figure 1:**
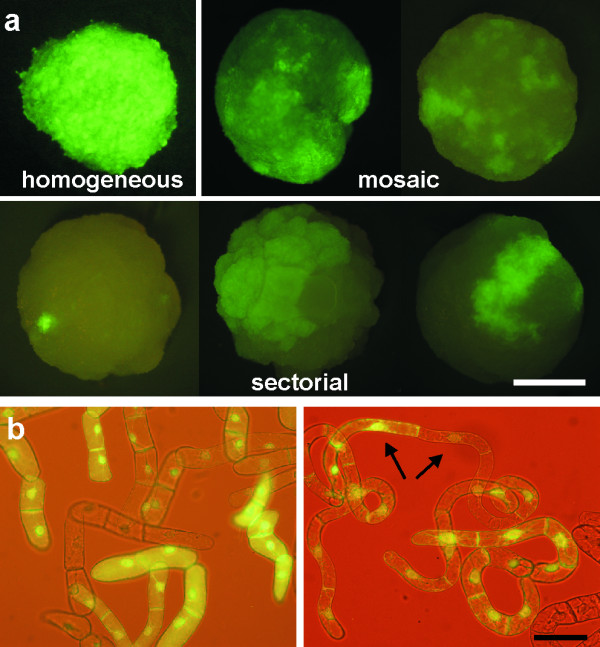
**Heterogeneity of GFP fluorescence in BY-2 *calli *and suspension cultures**. (**a**) Primary *calli *obtained after transformation, showing *calli *with homogeneous and heterogeneous *GFP *expression either in mosaic or sectorial arrangements of cell populations with distinct GFP fluorescence. (**b**) Non-homogenous *GFP *expression in suspension cells. The arrows indicate cells with evidently different *GFP *expressions located in a single file. Scale bars: 1 mm for A, 50 μm for B.

About 70% of round-shaped *calli *that were obtained after the *Agrobacterium*-mediated transformation of BY-2 cells displayed GFP fluorescence intensity sufficient for reliable evaluation of its homogeneity. Out of these *calli*, the GFP fluorescence was homogenous over the whole *callus *in only 35 – 50% cases in three independent transformations (Figure 1a; Table 1). The rest of the *calli *contained regions with evidently different levels of GFP fluorescence. Out of these heterogeneous *calli*, ~25% formed separate sectors (Figure 1a) and 36% were mixed in a mosaic arrangement (Figure 1a; Table 1). The frequency of these categories was comparable in all three transformations.

**Table 1:** Frequencies of BY-2 *calli *and suspensions with homogeneous and heterogeneous GFP fluorescence

		**Primary suspensions**			**Secondary suspensions**	
				
**GFP fluorescence in *callus***	**Primary *calli***	Homogeneous	Heterogeneous	**Secondary *calli***	Homogeneous	Heterogeneous
Homogeneous	39.3% ± 9.7%	29.2% ± 5.3%	70.8% ± 5.3%	93% ± 2.3%	46.3% ± 5.4%	53.7% ± 5.4%

Heterogeneous – mosaic	35.8% ± 13%	0%	100%	7% ± 2.3%	0%	100%

Heterogeneous – sectorial	24.9% ± 6.8%	0%	100%	0%	0%	100%

		**11.5% in total**	**88.5% in total**		**42.8% in total**	**57.2% in total**

Frequencies of homogeneous and heterogeneous *calli *and suspension cultures derived from these *calli *in primary lines obtained after transformation, and in secondary lines produced by cloning of primary heterogeneous (!) suspensions. Values represent means ± SD (n = 3); data are from three independent transformations; in every replication the number of evaluated lines was 60–80 for *calli *and ~20 for suspensions).

**Figure 2:**
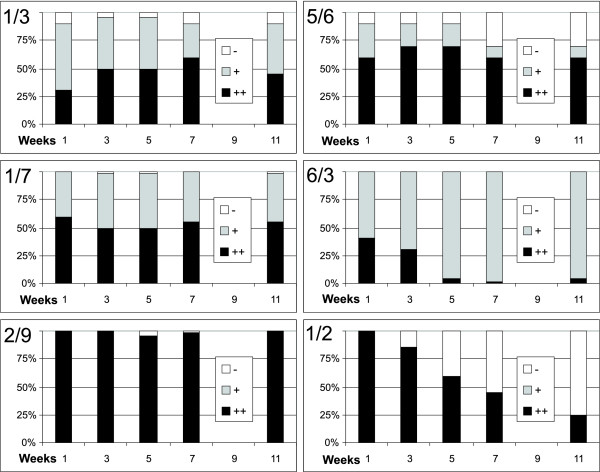
**Changes in frequency of GFP-expression categories in selected primary BY-2 suspension cultures with time**. GFP-expression categories: ++, strong fluorescence; + weak fluorescence, - no fluorescence.

Suspension cultures derived from the mixed *calli *contained cells with various GFP fluorescence intensities, as expected. However, also the majority (~70%) of homogenous *calli *gave rise to heterogeneous suspensions (Figure 1b). The cells with varied GFP fluorescence were predominantly in separate cell files, but occasionally were located even within a single cell file (Figure 1b). Classifying cells according to their GFP fluorescence intensities as high, low, or no fluorescence revealed that the proportions among the categories remained stable in the majority of the suspension cultures. Only in few lines (6/3; 1/2) the proportion of cells with high GFP fluorescence gradually declined with time (Figure 2).

### Cloning of suspension cultures

**Figure 3:**
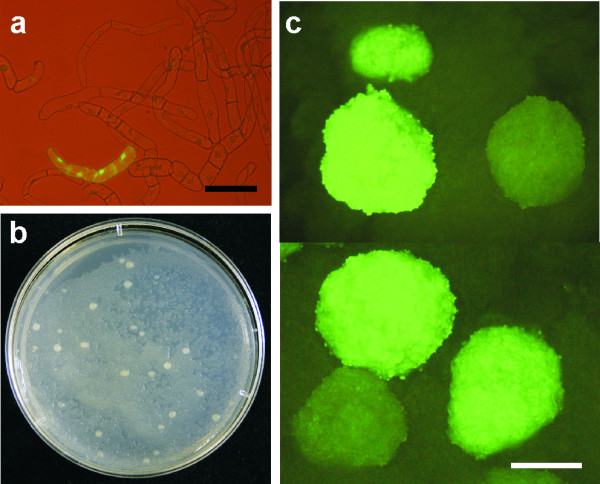
**A scheme of the BY-2 cloning procedure**. (**a**) Mixture of transgenic and wild-type lines before plating onto solid media. (**b**) Cloned *calli *emerging on the feeder layer ~10 days after plating. (**c**) Cloned calli of a heterogeneous line observed with a fluorescence stereomicroscope. Scale bars: 100 μm for A, 1 mm for C.

We introduced a simple and rapid method to generate clones from individual cells or cell files from the suspension cultures as follows: An excess of wild-type BY-2 suspension cells was added to the suspension culture of transformed (kanamycin resistant) cells in stationary phase of growth (Figure 3a). The mixture was then applied onto a Petri dish with solid MS medium containing kanamycin. Within 10 days, macroscopically visible *calli *appeared from individual resistant cells or cell files on the "feeder layer" of the wild-type BY-2 cells (Figure 3b, c). Few days later these "secondary" *calli *reached the size of 1 – 3 mm and could be transferred to a fresh medium for subsequent evaluation of GFP fluorescence homogeneity.

### GFP fluorescence in secondary *calli *and suspensions

**Figure 4:**
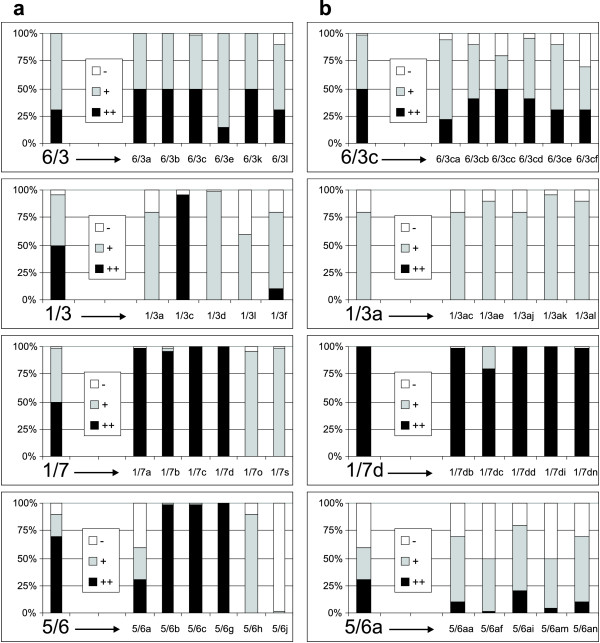
**Frequency of GFP-expression categories after the cloning of primary and secondary BY-2 lines**. (**a**) Primary cloning of suspensions obtained from *calli *directly after transformation. (**b**) Secondary cloning of selected subclones. The cloned lines are on the left, with progenies indicated by letters on the right. GFP-expression categories: ++, strong fluorescence; + weak fluorescence, - no fluorescence.

Cloning of suspension cultures with heterogeneous GFP fluorescence resulted in secondary *calli*, of which an average 93% gave rise to cell lines with almost exclusively homogenous GFP fluorescence (Table 1). Although the majority of secondary *calli *seemed to be homogenous, more than half of the suspension cultures derived from these *calli *still consisted of cell populations with various GFP levels (Table 1). The frequency of homogenous suspensions varied depending on the original suspension (Figure 4). Some lines (e.g. 1/3, 5/6) gave rise to both homogenous and heterogeneous suspension, whereas in the case of line 1/7 practically all the secondary and tertiary suspensions were homogenous, with either high or low GFP fluorescence intensities. In the case of line 6/3 or a secondary clone 5/6a, the original heterogeneity of GFP fluorescence persisted in all derived clones (Figure 4); even tertiary cloning of these "permanently heterogeneous" lines (e.g. 5/6am) did not diminish their heterogeneity. Generally, subsequent cloning of secondary clones produced almost exclusively homogeneous offspring. Their heterogeneity patterns in terms of proportions of individual GFP fluorescence categories corresponded to those of the original clones (Figure 4). Cloning of suspensions with homogeneous GFP fluorescence consistently gave homogenous subclones (e.g. 1/7d in Figure 4).

The proportions of GFP fluorescence categories in both hetero- and homogeneous suspensions remained stable for months in almost all cloned secondary suspensions (data not shown).

### Molecular analysis of the causes of GFP fluorescence heterogeneity

**Figure 5:**
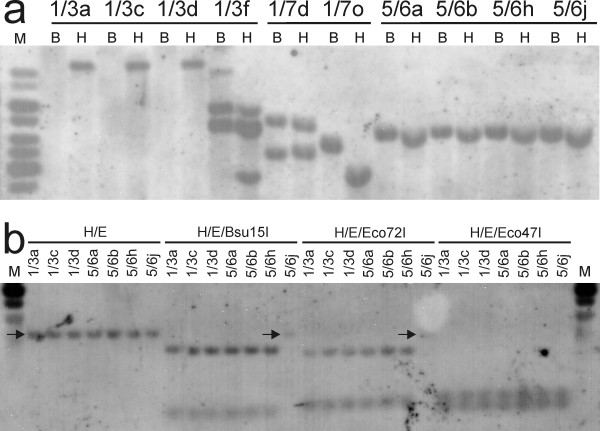
**Molecular analysis of selected primary clones**. (**a**) analysis of T-DNA insertions by Southern hybridization of total genomic DNA digested with either *Bam*HI (B) or *Hin*dIII (H). (**b**) Methylation analysis by Southern hybridization of total genomic DNA digested with *Hin*dIII (H) and *Eco*RI (E), cleaving out the *35S-GFP *cassette, and further with methylation-sensitive endocucleases *Bsu*15I, *Eco*72I or *Eco*47I, having restriction sites within the cassette; arrows indicate position of uncleaved *35S-GFP *cassette. The blots were hybridised with DIG-dUTP-labelled *GFP *probe. M, molecular weight ladder.

Analysis of T-DNA insertions in individual clones by Southern hybridisation (Figure 5) revealed that GFP fluorescence heterogeneity could have several causes. Lines 1/7 and 1/3 were composed of two genetically different clones that were separable by cloning, as shown by comparing 1/7d with 1/7o or 1/3f with other 1/3 clones (Figure 5). Other clones that also strongly differed in their proportions of the GFP fluorescence categories (Figure 4) seemed to be genetically identical, as shown by comparing 1/3a, 1/3c and 1/3d or 5/6a, 5/6b, 5/6h and 5/6j samples, where the *GFP *probe hybridised with equally-sized restriction fragments after cleavage with *Hin*dIII or *Bam*HI (Figure 5a). A possible presence of mutations within the *35S *promoter or *GFP *sequence was excluded by sequencing of *35S-GFP *cassettes obtained by PCR amplification; all sequences obtained from individual 5/6 clones 5/6a, 5/6b, 5/6h and 5/6j were identical (data not shown).

Genetically identical clones of lines 1/3 and 5/6 were further analysed with respect to DNA methylation. The *35S-GFP *cassette was cleaved out with *Eco*RI and *Hin*dIII, and exposed to the action of several methylation-sensitive enzymes (*Bsu*15I, *Eco*72I and *Eco*47I). Subsequent Southern blot analysis did not reveal any change in cytosine methylation at the analysed restriction sites, with the exception of the completely silenced clone 5/6j, whose DNA seemed to be methylated in the *Bsu*15I and *Eco*72I restriction sites (Figure 5b).

## Discussion

### Monitoring of GFP fluorescence – a suitable way to estimate *GFP *expression *in situ*

The green fluorescent protein (GFP) is an important reporter molecule for monitoring gene expression and protein localization *in vivo*, *in situ*, and in real-time observation. GFP fluorescence is stable, species-independent, and can be followed non-invasively in living cells where the green fluorescence reports active transcription and translation of the *GFP *gene [18]. Because of its simplicity, monitoring GFP fluorescence has been also routinely used in transgene silencing studies [19,20]. Although GFP is generally very stable [21], some differences in protein or fluorescence levels may occur due to protein degradation under certain treatments or in certain cell types. Nevertheless, in phenotypically homogeneous cell lines cultured under stable conditions, monitoring GFP fluorescence can be regarded as a suitable method for reliable estimation of *GFP *expression levels.

### Sources of *GFP *expression heterogeneity

Analysis of GFP fluorescence in primary suspensions obtained either directly from transformation or from secondary and tertiary clones revealed the coexistence of cell populations with different T-DNA insertions in some primary lines, representing genetic heterogeneity. Although, the majority of *GFP*-expression heterogeneity is most likely determined epigenetically.

#### Genetic heterogeneity

Southern hybridisation of genomic DNA isolated from selected clones of lines 1/7 and 1/3 clearly documented that even the round-shaped primary *calli *can contain cell populations with different T-DNA insertions. Because the probability of plating independently transformed cell files so closely together was low when considering plating density of ~30 – 50 *calli *per 6 cm-diameter plate, these cells are likely to represent the offspring of independently transformed cells that were located in a single cell file. Considering the large number of plated cells and transformation efficiency of ~0.1 – 0.5%, the results indicate that there could be cell files that are highly susceptible to *Agrobacterium*-mediated transformation. Alternatively, the genetically mixed *calli *could represent the offspring of a single cell transformed in S or G2 phase of the cell cycle with multiple T-DNAs, which then segregated unequally to daughter cells during mitosis.

#### Epigenetic heterogeneity

Analyses of genetically identical lines have documented developmentally- and environmentally-derived variability in transgene expression [10,17] and in stress-induced silencing [16]. In our study, *GFP *expression also varied among genetically identical clones even though the cells were phenotypically homogeneous and were cultured under stable conditions. This indicates that factors other than environmental or developmental or stress situations can induce changes in *GFP *expression. Gene expression is generally influenced by specific chromatin marks that may be present on both DNA and associated proteins [22]. Our methylation analysis of the *GF*P gene from selected genetically identical lines revealed that cytosins in analysed restriction sites were methylated only in some restriction sites and only in the line 5/6j with completely silenced *GFP *expression. This indicates that all other observed differences in *GFP *expression levels and heterogeneity are either independent of DNA methylation or methylation occurred in locations other than the selected restriction sites. The epigenetic state of chromatin is heritable through cell division, but can be easily modulated in response to certain triggers. For example, changes at the chromatin level such as cytosine methylation can accompany gene or transgene silencing [22] although the presence of methylated DNA is not necessarily related to the silenced phenotype [23].

Positional effect in the chromosomal environment at the site of transgene integration is known to influence transgene expression [13], although it seems to have much lower impact [11] than previously assumed [8]. Different sites, or arrangements, of T-DNA insertion can influence the accessibility/susceptibility of transgene to epigenetic regulation at either transcriptional or posttranscriptional level [15,24]; reviewed in [13]. Specifically in case of integration into heterochromatin region, the epigenetic information is almost regularly reflected in the chromatin structure of the inserted T-DNA, which results in transgene silencing [12,14,25]. In our experiments, *GFP *expression often varied strongly among clones with identical T-DNA insertions. For example, a single insertion resulted in completely different transgene expression patterns in genetically identical subclones of line 5/6 (Figure 4). In previous studies, transgene expression was analysed in clonal plant replicates generated long time after the integration event [10,17]. In contrast, the use of cell cultures and their cloning allowed us to analyse clones/replicates that arose immediately after the transgene integration. Since our results showed that *GFP*-expression patterns were stable and heritable in the cloned lines, the various expression patterns observed among genetically identical clones had to be established and stabilized in the offspring of the primary transformed cells early after the integration event. The process of integration of "naked" T-DNA is known to be accompanied by *de novo *establishment of specific chromatin composition and structure [25]. Our results clearly document that in certain insertion sites the establishment of different epigenetic states/transgene-expression patterns is accidental and independent of chromosomal environment in BY-2 cells.

Heterogeneity in GFP expression derived from the coexistence of either genetically or epigenetically different cells within the primary lines was in some cases resolved by cloning. However, in many clones (e.g. line 6/3; Figure 4) the heterogeneity in *GFP *expression among individual cells persisted and could not be resolved by subsequent cloning, representing a state of „permanent expression heterogeneity“. Van Leeuwen with colleagues also observed spatial and temporal variability in the expression of luciferase gene in different leaves and leaf sectors of stably transformed *Petunia *plants [10]. The authors attributed this mosaic character of transgene expression to temporal changes in the accessibility of promoter sequences for transcription factors, or variable levels of these factors in different leaf sectors at a time [10]. The pattern of this transgene expression variability was more or less specific for individual lines [10], similarly to the heterogeneity patterns observed in our lines 6/3c, 1/3a, 5/6a and their subclones (Figure 4). Since the heterogeneity patterns were heritable through subsequent cloning, they could be the result of specific variation in epigenetic states at a certain genomic locus [26].

Finally, the remaining evident cause of heterogeneity or instability of *GFP *expression is silencing [27]. Occurrence of transgene silencing was indicated by the presence of cells with contrasting GFP levels in a single cell file (Figure 1b) and by a gradual decline in the frequency of *GFP*-expressing cells observed in some lines (e.g. line 1/2; Figure 2). Silencing at the transcriptional level in connection with DNA methylation was demonstrated by the detection of methylated cytosin in clone 5/6j (Figure 5b). The role of methylation was confirmed by using the DNA-demethylation drug, 5-azacytidin [28], which reactivated *GFP *expression in several lines after several months of silenced *GFP *expression (e.g. lines 1/2, 5/6j; Nocarova and Fischer, unpublished). Silencing of transgene expression is naturally triggered mainly by high transcript levels [11], but may also be related to changes in the epigenetic status of plant genomic DNA in the process of dedifferentiation [29] that accompanies preparation of transgenic plants and plant cell lines.

### Cloning of plant cells – history and future

The first reports of cloning non-transgenic plant cells were published long time ago [30,31]. The method of cloning transgenic plant cell line introduced in our study has not been, to our knowledge, published and used before. Müller with colleagues described protoplast-based cloning of transgenic wheat lines, although this method was time-consuming and induced high frequency (up to 50% of clonal cells lines) of transgene silencing [16]. It indicates that the process of protoplast formation and regeneration may be accompanied by stress-induced epigenetic changes, causing transgene silencing [16]. The minimal occurrence of silencing in our experiments indicates that the drug selection of resistant cells during the cloning procedure causes significantly little stress. In contrast to protoplast-based cloning, our method does not always produce clones from single cells, because BY-2 cells remain temporarily attached in files. However, as the files originate from single cells, they are genetically homogeneous and *calli *derived from these files represent real clones. Although our cloning procedure did not, against expectations, lead exclusively to lines with homogenous *GFP *expression, clearly the cloning method is an effective way to substantially increase the number of homogenous lines. Whereas only ~10% of the primary cell lines were homogenous just after transformation, the cloning of largely heterogeneous lines produced additional 43% of homogeneous cell lines (Table 1).

In addition to generating homogeneously expressing transgenic lines, the cloning procedure appears to be a suitable tool for detailed analysis of the induction and stabilization of epigenetic changes connected with T-DNA insertion into the plant genomic DNA. Thus, in a modified arrangement, cloning of lines with silenced *GFP *expression using 5-azacytidin-containing media produced clones with reactivated *GFP *expression (Nocarova and Fischer, unpublished). Another possible use of the cloning procedure includes the cloning of epigenetically shifted lines habituated to certain conditions [32]. In transgenic lines carrying a negative selection gene whose expression is lethal under certain treatment, mixing an abundance of such a line with non-transformed line would enable cloning of reversed, non-transformed lines.

## Conclusion

By analysing GFP fluorescence in tobacco BY-2 cells, we found that expression of *GFP *transgene was highly heterogeneous in the majority of transgenic lines obtained directly from transformation. This heterogeneity had two causes: (1) genetic heterogeneity, namely the presence of cells with different T-DNA insertions; and (2) epigenetic heterogeneity, including transgene silencing, formation of stable epigenetic states early after transformation, and "permanent heterogeneity" with fluctuating changes in *GFP *expression. The genetic heterogeneity and the presence of cells in different but stable epigenetic states was responsible for almost half (43%) of the heterogeneity in the primary lines, and could be resolved by cloning. Because the cloning procedure can significantly increase the frequency/yield of homogenous lines, it is of high general impact for both molecular and biochemical analyses of BY-2 transgenic lines. In order to facilitate a simple way for assessment of transgene expression heterogeneity in both primary and cloned lines, it is highly advisable to use GFP-tagged transgenes. Alternatively, for transgenes that lack a visible, cell-autonomous phenotype the cloning procedure can be used to obtain genetically homogeneous lines with statistically higher chance of homogeneous transgene expression. Analysis of *GFP *expression in primary cell lines and their clones also showed that a single T-DNA insertion often resulted in various heritable transgene expression patterns/epigenetic states. These lines were probably established accidentally and became stabilized in the offspring of the primary transformed cells early after the integration event. Thus, the cloning procedure introduced in this study also appears to be suitable for analysing the sources of variability in transgene expression.

## Methods

### Cultivation and transformation of BY-2 cell line

Tobacco cell line BY-2 (*Nicotiana tabacum *L. cv. Bright Yellow 2 [33]) was cultured in modified MS medium [34]. Cells in suspension were subcultured every seventh day (1 ml of cells into 30 ml of liquid media). Stock BY-2 *calli *were maintained on media solidified with 0.7% (w/v) agar and subcultured monthly. The cultures were kept in darkness at 26°C; suspensions were placed on orbital incubator (IKA KS501, IKA Labortechnik, Staufen, Germany; orbital diameter 30 mm). Suspensions were prepared by resuspending of ~1 ml of fresh *calli *in 30 ml of liquid media by repeated pipetting through a cut tip (internal diameter ~5 mm).

Transformation of BY-2 line was performed by a slightly modified protocol introduced by [5]. A 2 ml aliquot of 3-day old BY-2 cells was co-cultivated with 200 μl of an overnight culture of *Agrobacterium tumefaciens *strain C58C1 carrying a helper plasmid *pGV2260 *[35] and a modified binary vector *pCP60 *[36] (kindly provided by dr. P. Ratet). The T-DNA contained a gene encoding red-shifted green fluorescent protein [37] (kindly provided by ABRC) inserted under the control of *CaMV 35S *promoter with a single enhancer region. The T-DNA further contained neomycin phosphotransferase gene (*NPTII*) driven by nopalin synthase promoter (*pNOS*), which provided kanamycin resistance. After co-cultivation, the cells were washed with 60 ml of 3% sucrose and 20 ml of liquid medium containing 100 μg/ml cefotaxim (CEFTAX, Hikma Farmaceutica, Terrugem, Portugal) in Nalgene filter holder (Nalgene, Rochester NY, USA). Thereafter, the cells were plated onto solid medium containing 50 μg/ml kanamycin and 100 μg/ml cefotaxim. Kanamycin-resistant colonies appeared after 3 to 4 weeks in darkness at 26°C. Transformed *calli *and suspensions were kept on media supplemented with kanamycin (50 μg/ml) for about two months and thereafter they were cultured as described for the BY-2 stock line.

### Assessment of *GFP *expression/fluorescence

Round-shaped primary *calli *in size of 1–3 mm (4 weeks after the transformation) were transferred onto fresh media. After additional 2 weeks of cultivation, the homogeneity of *GFP *expression was evaluated as a green fluorescence using a fluorescence stereomicroscope (Leica MZ16F). *Calli *containing a few sectors of different GFP fluorescence intensities separated by sharp borders were classified as mixed *calli *with a "sector arrangement". If the regions with different GFP fluorescence were mixed together without clear borders, or the number of separated regions was higher than approximately five, the arrangement was classified as "mosaic".

In suspension cultures, the homogeneity of *GFP *expression was evaluated using a fluorescence microscope Olympus Provis AX70 equipped with an FITC (U-MWU) filter set. The images were grabbed with a digital TV camera Sony DXC-950P (Sony Corp., Tokyo, Japan) and processed with Lucia image analysis software (Laboratory Imaging, Prague, Czech Republic). The proportions of cells with high, low, or no GFP fluorescence were estimated by evaluating ~100–150 cells. Only lines with clear difference between high and low expression categories are presented in the results section. A suspension was classified as homogenous if the portion of cells with minor classes of *GFP *expression level did not exceed 5% in total.

### Cloning of transgenic lines

Four weeks after transformation, the primary *calli *were transferred onto fresh solid medium containing kanamycin. After the next 3 weeks, the *calli *were gently resuspended in liquid medium and cultivated on a rotor shaker for a week. Thereafter, the suspension cells were subcultured (1.5 ml of suspension into 30 ml of fresh medium) and after additional 7 days when the culture reached stationary growth phase, the cells were used for cloning. The transgenic suspension culture was diluted with MS medium in a ratio 1:3 and mixed with 4 ml of similarly prepared wild-type stationary BY-2 culture in a ratio 1:1000. After gentle shaking, 500 μl of this mixture was evenly spread onto the Petri dish (∅ 6 cm) with solidified MS medium containing kanamycin. Clones of individual cells appeared as "secondary" *calli *(approximately 25 per plate) within two weeks.

### Molecular analysis

Total genomic DNA was isolated by Invisorb Spin Plant Mini Kit (Invitek, Berlin, Germany) from 100 mg (fresh weight) of filtered cells. Aliquots of 10 μg DNA were cleaved with *Hin*dIII and *Bam*HI (Fermentas, Burlington, Canada), which cleave the T-DNA in front and behind the *35S *promoter. For methylation analysis the *35S-GFP *cassette was cleaved out with *Hin*dIII and *Eco*RI, and thereafter the DNA was subjected to methylation-sensitive restriction enzymes that cleave within the *35S-GFP *cassette (*Bsu*15I, *Eco*72I, *Eco*47I; Fermentas), and separated on 0.8% agarose gel. Blotting was performed as described in [38]. Hybridisation with PCR-amplified probe of the whole GFP gene, labelled with DIG-dUTP (Roche Molecular Systems, Inc., Mannheim, Germany), was done according to manufacturer's instructions. Autoradiographic detection was done using chemiluminiscent substrate CDP-Star (Tropix, Bedford, USA). Fidelity of the insertions was confirmed by sequencing a PCR amplified *35S-GFP *cassette from total genomic DNA isolated from individual clones of cell line 5/6. PCR was done with *Pfu *polymerase according manufacturers instruction (Fermentas), sequencing was done by Sequencing laboratory, Faculty of Science, Charles University in Prague, Czech Republic).

## Authors' contributions

EN carried out all the experimental work and participated in manuscript writing. LF conceived the study, coordinated the experimental work and prepared the manuscript. Both authors read and approved the final manuscript.
